# Bacterial pericarditis presenting as hemorrhagic pericardial effusion in a 6-year-old girl

**DOI:** 10.4103/0974-2069.41062

**Published:** 2008

**Authors:** Anita Saxena, Navneet Singh, S. Ramakrishnan, Shyamsunder Kothari

**Affiliations:** Department of Cardiology, Cardiothoracic Centre, All India Institute of Medical Sciences, New Delhi, India

**Keywords:** Pericarditis, tamponade

## Abstract

Hemorrhagic pericardial effusion is known to occur due to tuberculosis, malignancy, uremia or trauma. We present a rare case of a 6 year old girl with bacterial pericarditis who had hemorrhagic pericardial effusion with cardiac tamponade.

## CASE REPORT

A 6-year-old girl presented with high-grade remittent fever and pain with swelling of the right knee joint for 10 days following a trivial blunt injury to the right knee. Two days later she developed retrosternal and epigastric pain, which increased during inspiration. On examination, she was found to be pale and febrile with an evidence of tachycardia and tachypnea. Her jugular venous pressure was elevated and she had pulsus paradoxus. She also had pedal edema and hepatomegaly. Auscultation revealed muffled heart sounds and a pleural rub.

Echocardiography demonstrated a large pericardial effusion with fibrinous strands and an early diastolic collapse of the right ventricle suggestive of tamponade physiology. An urgent pericardiocentesis was done. The fluid was hemorrhagic in nature with a hematocrit of 20%. The microscopic examination of the aspirate showed predominantly polymorphonuclear cells (85%); there were no malignant cells. The pericardial fluid was negative for bacterial growth. The levels of adenine deaminase (ADA), C3, ANA, and anti-dsDNA were normal. A PCR for *Mycobacterium tuberculosis* was also negative.

The routine hematological investigations showed anemia, polymorphonuclear leukocytosis, elevated hepatic enzymes, prolonged prothrombin time, and marginally impaired renal function. The X-ray of the right knee was normal. An ultrasound examination revealed joint effusion with evidence of polymyositis. Her computerized tomographic scan of the chest did not show any evidence of tuberculosis or lymphoma. A Mantoux test was negative.

Based on these findings, a provisional diagnosis of bacterial pericarditis with effusion was made despite the hemorrhagic nature of the fluid. She was started on broad-spectrum antibiotic with defervescence of fever in 96 h. A pigtail catheter was retained in the pericardial space and intermittent aspiration of the fluid was done. In view of the strong evidence of bacterial pericarditis with effusion, she was given 7 days of intrapericardial streptokinase to prevent constrictive pericarditis.[1] The pigtail catheter was removed when the aspirate diminished to negligible levels. She was discharged after 4 weeks of antibiotics along with traction, physiotherapy, and gradual mobilization of the right knee joint. At the time of discharge, she was afebrile, had no restriction of movements of the right knee, and had no evidence of constrictive pericarditis.

Two cases of hemorrhagic pericardial effusion are reported secondary to community-acquired pneumonia with *Chlamydophila pneumoniae*.[2] The diagnosis was made serologically. The pericardial fluid and serial blood cultures were negative, perhaps due to pre-hospitalization antibiotic therapy. The hemorrhagic pericardial effusion with *Staphylococcus aureus* pericarditis has been reported in a case where ruptured infective pseudoaneurysm of aortic arch was the source of hemorrhage.[3] Although we were unable to culture any organism from the fluid, there was strong evidence to suggest a bacterial etiology. This included an acute presentation, the polymorphonuclear predominance in the blood and pericardial fluid, and a rapid therapeutic response to antibiotics.

In conclusion, hemorrhagic pericardial effusion can rarely be seen secondary to bacterial pericarditis. However, a meticulous search must be made for other etiologies, which are far more common.
